# The effect of chronic endometritis and treatment on patients with unexplained infertility

**DOI:** 10.1186/s12905-023-02499-6

**Published:** 2023-06-30

**Authors:** Juan Gu, Qingqing Sun, Yujuan Qi, Fangfang Hu, Yijuan Cao

**Affiliations:** Reproductive Medical Center of Xuzhou Central Hospital, 221000 Xuzhou, China

**Keywords:** Chronic endometritis, Antibiotic treatment, Unexplained infertility, Pregnancy outcome

## Abstract

**Purpose:**

This paper was mainly conducted to investigate the effect of chronic endometritis (CE) on the clinical outcome of patients with unexplained infertility.

**Materials and methods:**

145 patients with unexplained infertility from the Reproductive Center of our hospital from January 2018 to December 2021 were selected as the unexplained infertility group. 42 patients with definite infertility causes were selected as the control group during the same period. Both groups of patients underwent hysteroscopy and immunohistochemical tests for CD38 and CD138. According to the results of hysteroscopy and immunohistochemistry, the incidence of CE between the two groups was analyzed. Patients with CE as CE group accepted oral antibiotic therapy for 14 days. Another 58 patients with unexplained infertility who did not undergo hysteroscopy and immunohistochemical tests for CD38 and CD138 were selected as the unexamined group. Both groups of patients were expected natural pregnancy. Follow-up lasted for 1 year, and the pregnant patients were followed up until delivery.The clinical pregnancy rate, spontaneous abortion rate and baby-carrying home rate of the two groups were compared.

**Results:**

There were 75 patients with CE in the unexplained infertility group, and the prevalence rate was 51.7% (75/145). Compared with the control group (28.6%), the incidence of CE was significantly higher (P < 0.05). After treated with antibiotic treatment, the patients’ clinical pregnancy rate was 61.3% (46/75) and baby-carrying home rate was 60% (45/75) in the CE group, which were higher than those in the unexamined group(43.1% & 36.2%) (P < 0.05), while the spontaneous abortion rate was 2.2% (1/46),which was lower than that in the unexamined group (16.0%) (P < 0.05).

**Conclusions:**

For patients with unexplained infertility, hysteroscopy combined with endometrial immunohistochemical detection of CD38 and CD138 should be performed in time to exclude CE. The clinical pregnancy outcome of CE patients can be significantly improved by antibiotic treatment.

## Introduction

CE is an inflammation characterized by infiltration of plasma cells in the endometrium. The clinical manifestations are nonspecific or even asymptomatic. However, CE can not only cause pain in the lower abdomen, abnormal uterine bleeding and increased vaginal secretions, but also affect the receptivity of the endometrium, resulting in repeated pregnancy loss, infertility and embryo implantation failure [1]. whether the uterine cavity is diseased, whether the uterine cavity shape is normal, whether the uterine cavity adhesion and endometrial state, etc can be directly observed by hysteroscopy. However, there are some limitations by hysteroscopy and is easily affected by the subjective factors of the surgeon. CD38 and CD138 are specific expression products of plasma cells. The detection rate of endometrial inflammatory lesions can be effectively improved by examing CD38 and CD138 in endometrial tissues [2]. Studies have shown that the diagnostic rate of chronic endometritis can be effectively improved by detecting the positive expressions of CD38 and CD138 in endometrial tissue combined with hysteroscopy [3]. Therefore, we studied the incidence of CE and its effect on clinical pregnancy outcome in patients with unexplained infertility through hysteroscopy combined with the detection of CD38 and CD138 in endometrial tissue.

## Materials and methods

### Materials

145 patients with unexplained infertility from the Reproductive Center of our hospital from January 2018 to December 2021 were selected as the unexplained infertility group and 42 patients with definite infertility causes were selected as the control group during the same period. Both groups of patients underwent hysteroscopy and immunohistochemical tests for CD38 and CD138. Patients with unexplained infertility diagnosed as CE were selected as CE group, and another 58 patients with unexplained infertility who didn’t accept hysteroscopy and immunohistochemical tests for CD38 and CD138 were selected as the unexamined group. Inclusion criteria: patients with unexplained infertility and those with clear infertility were younger than 35 years of age. Exclusion criteria: Ovulation disorders, endometriosis, intrauterine adhesions, hypoovarian function, infertility with internal and surgical complications, uterine malformations and organic lesions of the uterus, etc.

### Methods

1. Hysteroscopy and endometrial acquisition: The hysteroscopy and endometrial biopsy were performed after relevant preoperative examination at 3–7 day after menstruation. Endometrial biopsy plays a key role in the diagnosis of chronic Endometritis [4].The criteria for diagnosing CE under hysteroscopy [5] : (1) Endometrial congestion: dispersed or diffuse congestion, more obvious around the gland; (2) endometrial interstitial edema: the endometrial is still thickened and pale in the proliferation stage; (3) Endometrial polyps: fingerlike protrusions can be seen on the surface of the endometrial. A small curette was used to scratch the endometrium for pathological examination of suspected lesions.
2. Pathological examination and immunohistochemical tests for CD38 and CD138: The endometrial tissues were fixed with 10% formalin solution before being examined. The specimens were routinely dehydrated, embedded in paraffin, sliced and then stained with HE. The criterion for CE determination by HE staining is that typical plasma cells can be seen in the endometrial stroma [6]. Immunohistochemical tests for CD38 and CD138 were performed according to the instructions of the kit. After immunohistochemical staining, at 400 times of high magnification field of view, Five or more typical plasma cells with CD38 and CD138 positive plasma cells and CD38 and CD138 positive glandular epithelial cells found in the endometrial stroma were diagnosed as CE [7].
3. According to the results of hysteroscopy and immunohistochemistry, the incidence of CE in the two groups was analyzed.
4. Antibiotic treatment: Patients with CE were assigned as CE group and accepted oral antibiotic treatment twice a day for 14 days, doxycycline hydrochloride enteric-coated capsules, 20 capsules/box, 0.1g/ capsule (measured by doxycycline).
5. Comparison of clinical pregnancy outcomes: The paitients in CE group and the unexamined group were monitored for follicles and guided to have sex. The patients were followed up for 1 year, and the pregnant patients were followed up until delivery. The clinical pregnancy rate, spontaneous abortion rate and baby-carrying home rate of the two groups were compared.

### Statistical analysis

IBM SPSS Statistics 26 software was used for statistical analysis of the data. The measurement data were in line with normal distribution, expressed as ($\bar x \pm s$), and independent sample t test was used. The statistical data were expressed as n (%), and χ2 test or corrected χ2 test was used for comparison between groups. P < 0.05 was considered to be significant.

## Results

### Incidence of chronic endometritis in women with unexplained infertility

**Fig. 1:**
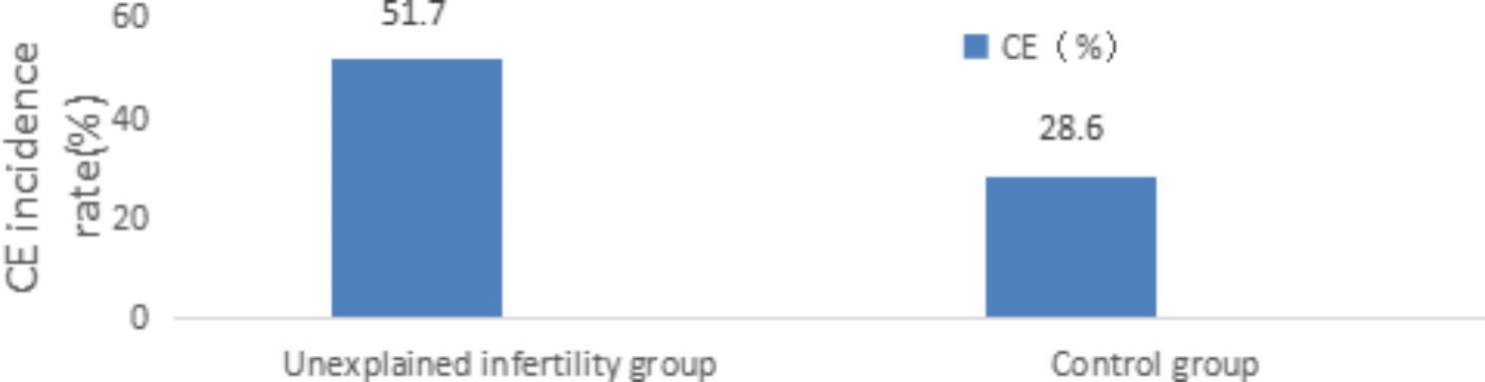
Comparison of the general situation between the CE group and the unexamined group

CD38 and CD138 were detected by hysteroscopy and immunohistochemistry in 145 patients with unknown infertility. The results showed that 75 patients with CE and 70 patients without CE, with a prevalence rate of 51.7%, which was significantly higher than that of the control group (28.6%), and the difference was statistically significant (P < 0.05).The results are displayed in Table 1 and Fig. 1

**Table 1:** Comparison of CE incidence between the two groups [n(%)]

	Unexplained infertility group(n = 145)	Control group (n = 42)	P
CE(%)	75(51.7)	12(28.6)	P = 0.008

There were no significant differences in age, infertility years, body mass index (IBM), follicle-stimulating hormone (FSH), Luteinizing hormone (LH )and anti-Müllerian hormone (AMH) between the two groups (P > 0.05), indicating that the two groups were comparable (Table 2).

**Table 2:** Comparison of clinical pregnancy rate, spontaneous abortion rate and baby-carrying home rate rate between the CE group and the unexamined group

	CE group(75)	unexamined group(58)	*P*
age(years old)	29.20 ± 2.89	28.95 ± 2.95	0.622
infertility years (year)	3.59 ± 1.37	3.26 ± 1.40	0.176
IBM(kg/m2)	21.74 ± 2.49	21.12 ± 2.15	0.134
FSH(mIU/ml)	6.77 ± 1.17	6.92 ± 1.18	0.482
AMH (ng/ml)	3.72 ± 1.41	3.90 ± 1.56	0.472

**Fig. 2:**
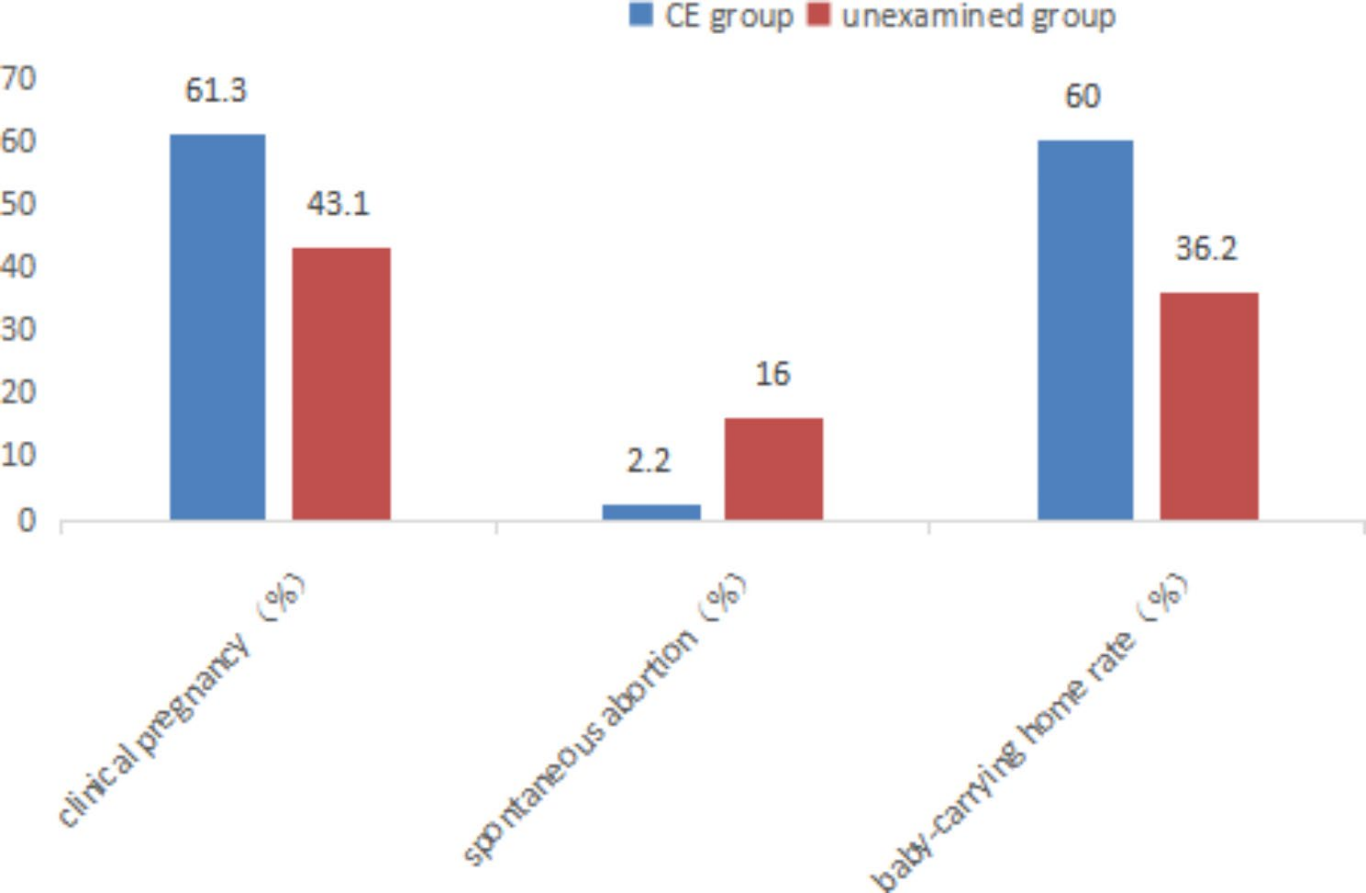
Comparison of clinical outcomes between CE group and unexamined group

The clinical pregnancy rate and baby-carrying home rate rate in CE group were 61.3% (46/75) and 60% (45/75) after antibiotic treatment higher than those of the unexamined group (41.3%&36.2%) (P < 0.05), and the spontaneous abortion rate of 2.2% (1/46) was lower than that of the unexamined group (18.2%) (P < 0.05).The results are displayed in Table 3 and Fig. 2

**Table 3:** Comparison of clinical outcomes between CE group and unexamined group [n(%)]

	CE group(75)	unexamined group(58)	P
clinical pregnancy (%)	46(61.3)	25(43.1)	0.037
spontaneous abortion(%)	1(2.2)	4(16.0)	0.049
baby-carrying home rate(%)	45(60.0)	21(36.2)	0.006

## Discussion

In recent years, the research about CE and infertility has attracted more and more attention from reproductive clinicians. It has been reported that the prevalence rate of CE in infertile patients is 2.8%~30% [8]. However, it was as high as 67.5% in patients with unexplained recurrent abortion or repeated embryo implantation failure [1]. Although the clinical treatment of infertility patients has greatly improved through cell and gene therapies, there are still some patients who experience repeated implantation failures [9].The study showed that mild CE had no effect on the IVF outcome, but women with severe CE had lower ongoing-pregnancy rate/live-birth-rate (OR 0.43, p = 0.003) and clinical-pregnancy rate (OR 0.40, p = 0.0007) [10].More and more studies believe that CE changes the endometrial microenvironment and thus affects endometrial receptivity, leading to the occurrence of adverse pregnancy.

It is easy to miss the CE diagnosis because there is no specific clinical and laboratory diagnosis method. At present, the commonly used detection methods include hysteroscopy, histopathology, microbial culture and so on. Because there is certain clinical subjectivity and limitations about the diagnosis of hysteroscopy, hysteroscopy combined with endometrial immunohistochemistry was adopted in this study to detect CD38 and CD138, which significantly improved the diagnosis rate of CE. This is consistent with Bouet’s views [11]. Bouet’s study showed that the sensitivity and specificity of hysteroscopy in the CE diagnosis were 40% (8/20) and 80%(59/74) and believed that hysteroscopy combined with endometrial biopsy was an effective means of CE diagnosis. Diagnosis and treatment of bacterial vaginosis, chronic endometritis, and pelvic inflammatory disease prior to attempted conception may be an important part of preconception care for symptomatic women to improve outcomes of natural and assisted reproduction [12]. Multiple embryo transfers, prolonged menstruation and in vitro fertilization-embryo transfer (IVF-ET) increased the risk of endometrial bacterial infection. Patients with tubal obstruction had an increased probability of secondary infection, which was related to endometrial inflammatory reaction and was a risk factor for CE [3, 13]. Therefore, the above patients should also be examined in time to rule out CE, and timely antibiotics should be given to those patients with CE, so as to improve the clinical pregnancy outcome.The transient, repeated and persistent impaired inflammatory state of the endometrium is a major factor of most problematic disorders in obstetrics/gynecology, such as endometrial polyps, unexplained infertility, miscarriage. The concept of impaired inflammatory state of the endometrium considers both infectious and non-infectious etiology and provids a newer approach to defective endometrial inflammation [14].

As for the treatment of endometritis, oral antibiotics are the commonly used treatment method at present. After treated with antibiotics, 82.3% of CE patients underwent re-histological examination, and the clinical pregnancy rate and live birth rate of cured patients were higher than those of uncured patients and those without CE detection [15]. Effective antibiotic treatment for chronic endometritis may improve the clinical pregnancy rate and live birth rate with unexplained recurrent pregnancy loss (RPL), and increase the ongoing pregnancy rate in patients with recurrent implantation failure [16].

A study of the effects of antibiotic therapy on obstetric outcomes in patients with RPL showed that live birth rates in patients with chronic endometritis improved after 14 days of antibiotic therapy compared to untreated chronic endometritis [17]. In this study, the clinical pregnancy rate and baby-carrying home rate rate of CE patients with unexplained infertility after antibiotic treatment were significantly higher than those of the unexamined group, while the spontaneous abortion rate was lower than that of the unexamined group.

The incidence of chronic endometritis is very high in patients with unexplained infertility. Timely diagnosis and treatment of chronic endometrial inflammation can significantly improve the natural pregnancy rate and baby-carrying home rate of patients. Patients with unexplained infertility experience a variety of examination and treatment failure, psychological pressure is greater. Timely searching for the causes of infertility and treatment, not only can improve the patient’s pregnancy outcome, but also greatly alleviate the patient’s mental anxiety, contribute to the stability of the family and society.

## Data Availability

The study results were included in this published article.

## Declarations

### Statement

All methods were carried out in accordance with relevant guidelines and regulations.

## Abbreviations

CE: chronic endometritis
IBM: Body mass index
FSH: Follicle-stimulating hormone
LH: Luteinizing hormone
AMH: Anti-Müllerian hormone
RPL: Recurrent pregnancy loss
IVF-ET: In vitro fertilization and embryo transfer

## References

[1] Kimura F, Takebayashi A, Ishida M (2019). Review: Chronic endometritis and its effect on reproduction [J]. J Obstet Gynaecol Res.

[2] Zargar M, Ghafourian M, Nikbakht R, Mir Hosseini V, Moradi Choghakabodi P (2020). Evaluating Chronic Endometritis in Women with Recurrent Implantation Failure and Recurrent Pregnancy Loss by Hysteroscopy and Immunohistochemistry. J Minim Invasive Gynecol.

[3] Li Xi-ya, Dong-mei ZHAO, Jie ZHANG (2022). Value of CD138 positive expression combined with hysteroscopy to the diagnosis of in vitro fertilization-embryo transfer repeated implantation failure complicated with chronic endometritis. J Chin Pract Diagn The.

[4] Vitale SG, Buzzaccarini G, Riemma G (2023). Endometrial biopsy: Indications, techniques and recommendations. An evidence-based guideline for clinical practice. J Gynecol Obstet Hum Reprod.

[5] Cicinelli E, Vitagliano A, Kumar A (2019). International Working Group for Standardization of Chronic Endometritis Diagnosis. Unified diagnostic criteria for chronic endometritis at fluid hysteroscopy: proposal and reliability evaluation through an international randomized-controlled observer study. Fertil Steril.

[6] Sfakianoudis K, Simopoulou M, Nitsos N (2019). Successful Implantation and Live Birth Following Autologous Platelet-rich Plasma Treatment for a Patient with Recurrent Implantation Failure and Chronic Endometritis. Vivo.

[7] Adegboyega PA, Pei Y, McLarty J (2010). Relationship between eosinophils and chronic endometritis. Hum Pathol.

[8] Kitaya K, Takeuchi T, Mizuta S (2018). Endometritis: new time, new concepts [J]. Fertil Steril.

[9] Medenica S, Abazovic D, Ljubić A et al. The Role of Cell and Gene Therapies in the Treatment of Infertility in Patients with Thyroid Autoimmunity. Int J Endocrinol. 2022, 4842316. 10.1155/2022/4842316 PMC9448571 36081621

[10] Vitagliano A, Laganà AS, De Ziegler D (2022). Chronic Endometritis in Infertile Women: Impact of Untreated Disease, Plasma Cell Count and Antibiotic Therapy on IVF Outcome-A Systematic Review and Meta-Analysis. Diagnostics (Basel).

[11] Bouet PE, El Hachem H, Monceau E (2016). Chronic endometritis in women with recurrent pregnancy loss and recurrent implantation failure: prevalence and role of office hysteroscopy and immunohistochemistry in diagnosis. Fertil Steril.

[12] Ravel J, Moreno I, Simón C (2021). Bacterial vaginosis and its association with infertility, endometritis, and pelvic inflammatory disease. Am J Obstet Gynecol.

[13] Martingano D, Renson A, Rogoff S (2019). Daily gentamicin using ideal body weight demonstrates lower risk of postpartum endometritis and increased chance of successful outcome compared with traditional 8-hour dosing for the treatment of intrapartum chorioamnionitis. J Matern Fetal Neonatal Med.

[14] Drizi A, Djokovic D, Laganà AS (2020). Impaired inflammatory state of the endometrium: a multifaceted approach to endometrial inflammation. Current insights and future directions. Prz Menopauzalny.

[15] Cicinelli E, Matteo M, Trojano G (2018). Chronic endometritis in patients with unexplained infertility: Prevalence and effects of antibiotic treatment on spontaneous conception. Am J Reprod Immunol.

[16] Puente E, Alonso L, Laganà AS (2020). Chronic Endometritis: Old Problem, Novel Insights and Future Challenges. Int J Fertil Steril.

[17] Gay C, Hamdaoui N, Pauly V, Rojat Habib MC, Djemli A, Carmassi M, Chau C, Bretelle F (2021). Impact of antibiotic treatment for chronic endometritis on unexplained recurrent pregnancy loss. J Gynecol Obstet Hum Reprod.

